# Leishmania kinetoplast DNA contributes to parasite burden in infected macrophages: Critical role of the cGAS-STING-TBK1 signaling pathway in macrophage parasitemia

**DOI:** 10.3389/fimmu.2022.1007070

**Published:** 2022-11-02

**Authors:** Ismail Cem Yilmaz, Emre Dunuroglu, Ihsan Cihan Ayanoglu, Emre Mert Ipekoglu, Muzaffer Yildirim, Nogay Girginkardesler, Yusuf Ozbel, Seray Toz, Ahmet Ozbilgin, Gamze Aykut, Ihsan Gursel, Mayda Gursel

**Affiliations:** ^1^ Basic and Translational Research Program, Izmir Biomedicine and Genome Center, Izmir, Turkey; ^2^ Department of Biological Sciences, Middle East Technical University, Ankara, Turkey; ^3^ Molecular Biology and Genetics Department, Bilkent University, Ankara, Turkey; ^4^ Department of Parasitology, School of Medicine, Celal Bayar University, Manisa, Turkey; ^5^ Department of Parasitology, Ege University, Izmir, Turkey

**Keywords:** leishmania, kinetoplast DNA (kDNA), cGAS, STING, TBK1, amlexanox, 2'3'-cGAMP, H151

## Abstract

Leishmania parasites harbor a unique network of circular DNA known as kinetoplast DNA (kDNA). The role of kDNA in leishmania infections is poorly understood. Herein, we show that kDNA delivery to the cytosol of *Leishmania major* infected THP-1 macrophages provoked increased parasite loads when compared to untreated cells, hinting at the involvement of cytosolic DNA sensors in facilitating parasite evasion from the immune system. Parasite proliferation was significantly hindered in cGAS- STING- and TBK-1 knockout THP-1 macrophages when compared to wild type cells. Nanostring nCounter gene expression analysis on *L. major* infected wild type versus knockout cells revealed that some of the most upregulated genes including, Granulysin (GNLY), Chitotriosidase-1 (CHIT1), Sialomucin core protein 24 (CD164), SLAM Family Member 7 (SLAMF7), insulin-like growth factor receptor 2 (IGF2R) and apolipoprotein E (APOE) were identical in infected cGAS and TBK1 knockout cells, implying their involvement in parasite control. Amlexanox treatment (a TBK1 inhibitor) of *L. major* infected wild type cells inhibited both the percentage and the parasite load of infected THP-1 cells and delayed footpad swelling in parasite infected mice. Collectively, these results suggest that leishmania parasites might hijack the cGAS-STING-TBK1 signaling pathway to their own advantage and the TBK1 inhibitor amlexanox could be of interest as a candidate drug in treatment of cutaneous leishmaniasis.

## Introduction

Leishmaniasis is a vector-borne neglected disease caused by protozoan parasites belonging to the Trypanosomatidae family, Kinetoplastida order, and Leishmania genus (1–6). The disease is considered to cause the ninth largest disease burden among infectious diseases (7–9) and has been listed as the second most common parasitic infection worldwide (10). Over 20 different leishmania species can be transmitted to humans during a blood meal of infected female phlebotomine sandflies (7, 11). Within the insect midgut, the parasites undergo several developmental changes and differentiate into infectious metacyclic promastigotes which are then disseminated into the mammalian host during a blood meal and are rapidly phagocytosed by mono and polymorphonuclear (PMN) phagocytes. Depending on the infecting Leishmania species and the host’s immune status, the disease manifests as cutaneous, mucocutaneous or visceral leishmaniasis (9, 12–17).

Leishmania species share a digenetic life cycle, inhabiting an insect vector and a vertebrate host. The parasites grow as motile, flagellated extracellular promastigotes in the sandfly midgut, ultimately differentiating into infectious metacyclic promastigotes (18–21). Following entry into the mammalian host, parasites rapidly localize and invade the phagocytes. Within macrophages, the parasites reside in phagolysosomal parasitophorous vacuoles, transform into non-motile, non-flagellated amastigotes and multiply by binary fission (22, 23). Macrophages are equipped with multiple germline encoded pattern recognition receptors (PRRs) that recognize pathogen-associated molecular patterns (PAMPs) originating from pathogens. In this context, the cytosolic DNA sensor cyclic GMP-AMP synthase (cGAS), its downstream adaptor stimulator of interferon genes (STING) and the kinase TBK1 constitute a fundamental pathway critical for the outcome of infections (24–26).

Emerging evidence suggests that cGAS-STING cytosolic DNA sensing pathway contributes to pathogenesis of certain parasitic infections (26–30). With respect to leishmania, whether the cGAS/STING/TBK1 signaling plays a protective or a pathological role is unknown.

Trypanosoma and leishmania parasites harbor one of the most unusual DNA structures in nature. The mitochondrial DNA of these parasites (the kinetoplast DNA (kDNA)) consist of a single network of interlocked circular DNAs composed of 25–50 maxicircles and 5,000–10,000 of minicircles (31–34). Approximately 30% of parasite DNA is of kinetoplast origin (35). Interestingly, kDNA persists in tissues of Chagas patients and presence of kDNA correlates with inflammatory cell infiltration (36). Based on this observation, it is possible that kDNA-induced immune recognition might alter the immune response during an infection.

Herein, we assessed the effect of kDNA on parasite loads in *in vitro* infection models and evaluated the role of cGAS-STING-TBK1 DNA-sensing pathway in the context of *L. major* infection. Our findings showed that stimulation of immune cells with kDNA increased parasite loads *in vitro*. We further showed that absence of cGAS, STING or TBK1 proteins significantly diminished parasite loads of differentiated THP-1 macrophages, implicating a role of this DNA sensing pathway in pathogenesis of leishmania infections.

## Materials and methods

### Parasites

*L. major* parasites (whom/tr/2014/cbu33) used in this study were isolated from a cutaneous lesion of a patient. The species of the isolated parasite was identified and validated (37). The isolated parasites were subjected to *in vivo* passaging in BALB/c mice to maintain their virulence.

### Parasite culture

*L. major* promastigotes were maintained and axenically cultured in plug sealed tissue culture flasks (5-6 x 10^6^ cells/ml) in RPMI 1640 growth medium (20 mM HEPES, 100 units/ml penicillin, 100 µg/ml streptomycin, 20% (v/v) heat inactivated FBS) at 26°C inside a standard incubator without CO_2_. Parasites were sub-cultured at late-logarithmic phase (≈25x10^6^ parasites/ml) by diluting the culture medium with fresh growth medium. Axenic promastigotes were passaged to a maximum of 12 passages (≈36 days) to prevent loss of virulence.

### Kinetoplast DNA isolation

Kinetoplast DNA (kDNA) from *L. major* were isolated as previously described (38) with modifications. 10^9^ parasites were sedimented at 3,000g for 10 minutes at 4°C. The pellet was resuspended in 6-8 ml NET buffer (100 mM NaCl, 10 mM EDTA (pH:8.0),10 mM Tris HCl (pH:8.0)) and mixed vigorously. The suspension was centrifuged once more. Pellet was resuspended in NET buffer. Subsequently, Proteinase K and Sarcosyl were added into the solution at final concentrations of 100 μg/ml and 1% (v/v), respectively. The mixture was incubated overnight for completion of cell lysis.

Following incubation, the lysate was centrifuged at 24,000g for 2 hours at 4°C to sediment the kDNA networks. Pellet was resuspended in NET buffer and kDNA suspension was treated with Plasmid-Safe™ ATP-Dependent DNase and RNase A. Next, one volume of phenol pH:7.9 was added onto the solution and mixed by inversions until an emulsion formed. The emulsion was centrifuged at 14,000g for 90 seconds at RT. Following centrifugation, aqueous phase was transferred to a new tube. One volume of chloroform was added onto the separated aqueous phase and the abovementioned mixing and centrifugation steps were repeated. Then, 1/3 volume of 5 M ammonium acetate solution was added onto the collected aqueous phase, followed by addition of 2-3 volumes of molecular biology grade absolute ethanol and mixing. Samples were incubated at -20°C overnight. After incubation, samples were centrifuged at maximum speed of a standard minicentrifuge and supernatants were decanted. Precipitate was washed once more with 70% ethanol, air-dried and dissolved in DNAse-free ultra-pure water. DNA concentrations of isolates were measured by using NanoDrop Microvolume Spectrophotometer.

### Conformational analysis of kDNA by atomic force microscopy

kDNA isolates were diluted in DNA attachment buffer, pH:8.0 (40 mM HEPES-Cl,10 mM MgCl_2_) at a final concentration of 50 μg/ml. Mica discs were immobilized on metal discs. Approximately 50 μl of kDNA in the DNA attachment buffer was added onto freshly cleaved mica disc surface and incubated at RT for 5 minutes. Then, the excess buffer was rinsed with ultra-pure water and the sample was air-dried. Air-dried samples were scanned on an Ambient AFM (Nanomagnetics Instruments, UK) following the manufacturer’s instructions. Briefly, laser and detector positions were arranged to obtain a total force of 3.0 V with no lateral and nominal forces. Root mean square (RMS) value was set around 2-2.2. Oscillation amplitude was set to half of RMS value and samples were scanned at non-contact mode by using the NMI SPM (v2.0.37) software. Scanned images were plane-corrected and scaled by using the NMI Viewer (v1.4) software.

### Generation of CFSE-labeled parasites

Metacyclic parasites were resuspended in DPBS at a final concentration of 5x10^6^ cells/ml. Carboxyfluorescein Succinimidyl Ester (CFSE) was added onto parasite suspension at a final concentration of 5 μM and incubated in dark for 20 minutes at RT. Following washing in DPBS once, resulting pellet was resuspended in Leishmania Infection Medium (RPMI 1640 supplemented with 2%(v/v) FBS, 20mM HEPES, 100 units/ml penicillin, 100µg/ml streptomycin).

### Infection of PMA-differentiated THP-1 cells with CFSE labelled parasites in the presence of kDNA and gDNA

Phorbol myristate acetate (PMA) differentiated THP-1 cells (10^5^ cells/well) were infected with CFSE-labeled metacyclic parasites (10^6^ parasites/well) in Leishmania Infection Medium to achieve a multiplicity of infection (MOI) of 1:10 (THP-1:parasites). kDNA complexed with Lipofectamine 2000 were added onto corresponding leishmania-THP-1 mixtures to reach final DNA concentrations of 0.25, 1 and 4 μg/ml. Samples were incubated at 37°C with 5% CO_2_ for 24 hours. Cells were then washed with pre-warmed null RPMI 1640 medium thrice, incubated with 50 μl 1X Trypsin-EDTA solution for 10-15 minutes and detached cells were collected and transferred to new microcentrifuge tubes. 500 μl 10% Regular RPMI 1640 medium was added onto cells to inactivate trypsin. Samples were centrifuged at 1,500g for 5 minutes and pellets were resuspended in 10% Regular RPMI 1640 Medium. Resuspended samples were acquired on Accuri C6 cytometer (Becton, Dickonson, U.S.A.). PMA-differentiated THP-1 cells were gated with respect to FSC-SSC parameters. Infection rates and MFI values were determined based on CFSE emission.

### Fluorescence microscopy analysis of leishmania infected THP-1 Cells

PMA-differentiated THP-1 cells were infected with unlabeled metacyclic parasites and stimulated with kDNA. Following 24h incubation, media were aspirated from wells. DPBS containing 1 μM SYTO^®^ 16 Green fluorescent nucleic acid stain (permeable) and 5 μM SYTOX^®^ Orange nucleic acid stain (impermeable) were added into the wells. Cells were incubated at 37°C for 20 minutes with staining solution. Then, staining solution was aspirated and cells were washed by PBS once. 10% Regular RPMI 1640 Medium was added onto cells and infected cells were observed on green and red channels of FLoid Cell Imaging Station and merged images from each channel were taken from different regions of the wells.

### Generation of eGFP expressing transgenic *L. major*

Plasmid pLEXSY-neo2.1 (Jena Biosciences, Germany) was custom-designed to encode codon optimized EGFP-LUCIFERASE (EGFP-LUC) fusion sequence. For generating transgenic parasites, 10 μg of pLEXSY EGFP-LUC vector was digested with 10 units of SwaI at RT for 2 hours. Samples were run on 0.8% (w/v) agarose gel for confirmation of successful digestion. Parasites at log phase (~15x10^6^ parasites/ml) were centrifuged at 1,500g for 10 minutes at RT. Pellet was resuspended in DPBS, washed once and 4x10^7^ were re-suspended in 400 μl of ice-cold electroporation buffer. 10 μg linearized expression plasmid was transferred onto parasites in the electroporation buffer and the mixture was kept on ice. DNA-parasite mixture was transferred to Gene Pulser electroporation cuvettes with 0.2 cm gap (Biorad, U.S.A.). Samples were pulsed for 30 seconds on the Gene Pulser^®^ II Electroporation System (Biorad, U.S.A.) using the following parameters: 750 V (3750V/cm), 25 μF and 200 Ω. Pulsing was repeated twice using identical parameters and with 30 second breaks between pulses. Electroporated samples were incubated on ice for 10 minutes. After incubation, parasites were transferred to a T25 plug seal tissue culture flask filled with pre-warmed (25°C) 4 ml Leishmania growth medium. Parasites were allowed to rest for 24 hours. 24 hours after the electroporation, 50 μg/ml of Neomycin was used as a selection marker to enrich EGFP-LUC positive parasites.

### *In vitro* infection of THP-1 cell lines with eGFP expressing *L. major*

THP1-Dual™ WT, THP1-Dual™ KO-STING, THP1-Dual™ KO-cGAS, THP1-Dual™ KO-TBK1 cell lines were cultured in 10% (v/v) FBS supplemented RPMI 1640 medium containing 100 μg/ml zeocin and 10 μg/ml blasticidin (*In vivo*gen, France). For macrophage differentiation of THP-1 Dual cells, PMA was used at high concentration (50ng/ml) overnight or low concentration (5ng/ml) for 48 hours. After PMA treatment, to equalize the initial macrophage number for all cell lines, cells were detached by using accutase solution (Biolegend, U.S.A.), counted on Novocyte 2060R flow cytometer (ACEA Biosciences, U.S.A.) and distributed to 96-well plates at a density of 1x10^5^ cells/well in Leishmania Infection Medium (RPMI 1640 supplemented with 2% FBS, 20mM HEPES, 100 units/ml penicillin, 100µg/ml streptomycin). Multiplicity of infection (MOI) was optimized as 1:10 (THP-1:stationary phase *L. major*). After 6h of infection, wells were washed with DPBS to remove excess parasites and fresh 10% (v/v) FBS supplemented RPMI 1640 medium was added on to the infected THP-1 cells. Percent infection rates and parasite loads (MFI) were based on the eGFP signal and were quantitated on a Novocyte 2060R flow cytometer (ACEA Biosciences, U.S.A.). In certain experiments, Amlexanox (*In vivo*gen, France) was added into the THP-1 growth medium 1 hour prior to infection at indicated concentrations or 24 hours post-infection.

### Total RNA isolation from infected THP-1 cells

THP1-Dual™ WT, THP1-Dual™ KO-cGAS and THP1-Dual™ KO-TBK1 cells were differentiated overnight in RPMI 1640 media supplemented with 10% (v/v) FBS and 50 ng/ml PMA in 6-well tissue culture plates at a density of 2.7x10^6^ cells/well. *In vitro* infection was performed by co-incubating stationary phase (2-3 days after reaching late-logarithmic phase) eGFP expressing *L. major* parasites with THP-1 cell lines at a MOI of 1:10 (macrophage:parasite) for 6 hours in Leishmania Infection Medium (RPMI 1640 supplemented with 2% FBS, 20mM HEPES, 100 units/ml penicillin, 100µg/ml streptomycin). The incubation period was followed by three DPBS washes to remove extracellular *L. major* parasites and replenishment of media. At 48 hours post-infection, supernatants were aspirated, and 1 ml of RiboEx™ was added onto each well. After cellular material was collected, total RNA isolation was performed following the manufacturers’ instructions and the RNA Clean & Concentrator™-25 (Zymo Research, U.S.A.) kit.

### Differential gene expression analysis of THP-1 cells

Following quantification of RNA samples and assessment of their purity (A260/A280 and A260/A230) using NanoDrop™ spectrophotometer, 50 ng of RNA sample for each group was processed with nCounter PanCancer Immune Profiling CS Kit (no MK) (NanoString Technologies, Inc., U.S.A.) according to the manufacturer’s instructions. Obtained raw data was analyzed using nSolver Analysis software (v4, NanoString Technologies, Inc., U.S.A.) and nCounter^®^ Advanced Analysis Software (v2, NanoString Technologies, Inc., U.S.A.).

### Amlexanox therapy for the *L. major* infected mice

All protocols carried out in this study were approved by animal ethics committee of Bilkent University (Protocol No: BILAEC-2019/44). A total of seven mice were injected with 50 μl of 9x10^6^ metacyclic *L. major* parasites in DPBS in their left footpads using a 1 ml syringe with a 26G needle. 41 days post-infection, 50 μl of 10 mg/ml amlexanox (n=4) or 50 μl of DMSO control (n=3) was injected into mice intraperitoneally for 12 consecutive days. A digital caliper was used for lesion surveillance by measuring both the depth and width of the footpads.

### Statistical analyses

Statistical tests were performed using Graphpad Prism (v9.) unless specified otherwise Specific statistical tests used for each comparison were stated in figure legends. Asterisks represent significant p-values as detailed below.


ns=not significant, *:p≤0.05, **p≤0.01, ***p≤0.001, ****:p≤0.0001

## Results

### Delivery of kDNA into cytosol increases parasite burden in infected macrophages

Although cGAS binds dsDNA in a sequence-independent manner, bent DNA was shown to favor dimerization and facilitate nucleation of cGAS-DNA ladder formation (39). Interestingly, bent DNA helices were first described in the kinetoplast DNA minicircles (40). Furthermore, kDNA persistence in tissues of infected hosts correlates with inflammatory cell infiltrates (36), suggesting a role for this unusual DNA structure in pathogenesis of infections caused by protozoan parasites of the order Kinetoplastida.

**Figure 1:**
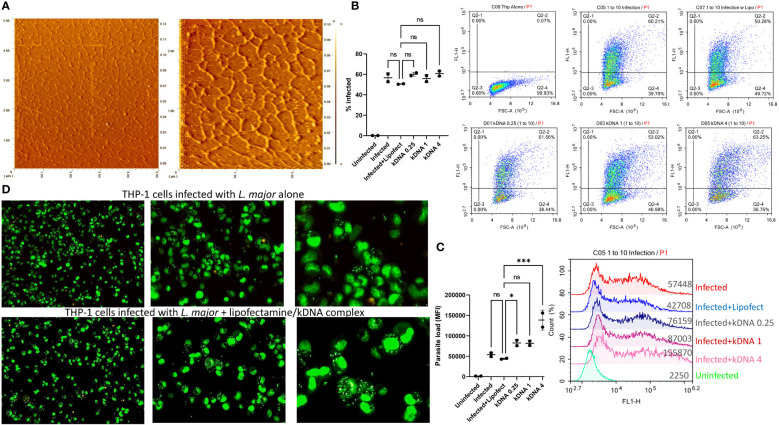
Effect of Cytosolic kDNA delivery on *in vitro* Leishmania infection. **(A)** AFM images of the purified kDNA networks with scanning areas of 5x5 μm (left) and 2.5x2.5 μm (right). PMA-differentiated THP-1 cells (5x10^5^ cells/ml) were incubated with CFSE labeled parasites (5x10^6^ parasites/ml) for 24 hours in the presence or absence of kDNA. Percentage of infected cells **(B)** and total parasite loads **(C)** were evaluated by flow cytometry. Numbers on x-axis labels indicate concentration of kDNA (μg/ml). Data represent the average of two independent experiments ± SD. All groups were compared statistically by one-way ANOVA followed by Dunnet`s multiple comparison test. **(D)** Fluorescence microscopy images of infected, SYTO Green stained THP-1 cells (from left to right, 10X 20X and 40X) untreated (upper panels) or treated with 1 μg/ml kDNA. Images are representatives of two independent experiments. ns=not significant, *p≤0.05, **p≤0.01, ***p≤0.001, ****p≤0.0001

To assess whether Leishmania kDNA contributes to disease progression, we first isolated kDNA from the parasites. For this, kDNA was purified from late-log phase parasites and treated with Plasmid-Safe™ ATP-Dependent DNase and RNase A to eliminate residual genomic DNA and RNA contaminants in the kDNA fraction, respectively. Following purification, isolated kDNA networks were analyzed using atomic force microscopy (AFM). 5x5 and 2.5x2.5 µm scans demonstrated the typical kDNA network structure composed of interlocked circular DNAs (**Figure 1A**), confirming the success of the isolation protocol.

Next, PMA differentiated THP-1 macrophages were infected with CFSE labeled parasites in the presence or absence of different concentrations of kDNA and percentage of infected cells and total parasite loads (based on MFI) were evaluated by flow cytometry (**Figures 1B, C**). Percentage of infected THP-1 cells did not change significantly in the presence of kDNA (**Figure 1B**) but the parasite load was significantly increased in the presence of the highest dose of kDNA (**Figure 1C**). Of note, use of lipofectamine had no adverse effect on infected cell percentage or parasite loads when compared to cells infected in the absence of lipofectamine (**Figures 1B, C**), indicating that the kDNA-induced increased parasite load was not an artifact caused by the inclusion of the transfection reagent. Furthermore, treated THP-1 cells were responsive to kDNA delivery as evidenced by type I interferon production (**Supplementary Figure 1**), suggestive of activation of a cytosolic DNA sensing pathway. Of interest, kDNA was a more potent stimulator of type I interferon production when compared to stimulation with an equal concentration of linear Leishmania genomic DNA (**Supplementary Figure 1**).

THP-1 macrophages infected with unlabeled *L. major* in the absence or presence of kDNA (1 μg/ml) were also stained with the membrane permeable dye SytoGreen 16 and imaged under a fluorescence microscope. kDNA transfected cells showed higher number of parasites (**Figure 1D**, lower panel) per cell when compared to the untreated group (**Figure 1D**, upper panel).

These results suggested that cytosolic delivery of kDNA might contribute to parasitaemia in infected macrophages, indicating a role for cytosolic DNA sensors in modulating the response to leishmania infection.

### cGAS, STING and TBK1 knockout THP-1 cell lines are resistant to leishmania infection

**Figure 2:**
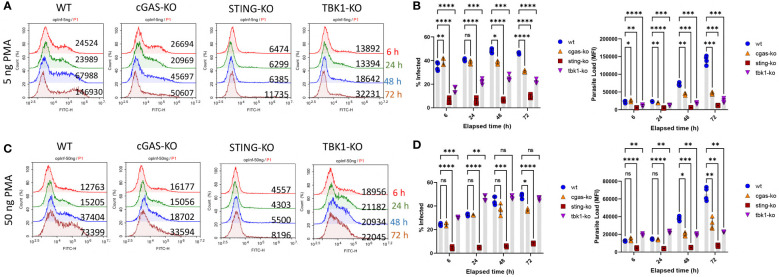
Kinetics of Leishmania infection in WT versus cGAS-, STING- or TBK1-knockout THP-1 cells. Low dose [**(A)** and **(B)** or high dose **(C)** and **(D)**] PMA differentiated THP-1 cells were infected with eGFP expressing *L. major* parasites at a MOI of 1:10 (macrophage:parasite). At indicated time points (6,24,48 and 72 h), infection percentages and parasite loads were quantified using a flow cytometer. Data presented in graphs represent the average of two independent experiments with technical replicates ± SD. Statistical comparisons to WT cells were based on two-way ANOVA followed by Tukey’s multiple comparison test. ns, not significant, *p≤0.05, **p≤0.01, ***p≤0.001, ****p≤0.0001.

To investigate the role of cytosolic DNA sensing pathway on leishmania infection rates, three different THP-1 cell lines (cGAS, STING and TBK1 knockout) were infected with eGFP expressing transgenic parasites in parallel with wild type (WT) THP-1 cells. Since PMA concentration used for macrophage differentiation may impact the maturation level of THP-1 cells and therefore the initial phagocytic activity of the macrophages, infection experiments were conducted using cells differentiated with a low (5 ng/ml) and a high (50 ng/ml) dose of PMA. When low-dose PMA differentiated cells were used, percent infected cells (**Supplementary Figure 2** and **Figure 2B**) and parasite loads (**Figures 2A, B**) of WT cells were consistently higher than the knockout cells throughout the 3-day infection period. However, for the STING and TBK1 knockout cells, the initial phagocytic capacities (at 6h) were significantly lower than the WT cells (**Supplementary Figure 2**; **Figures 2A, B**) and hence the observed lower infection rates might have stemmed from fewer infecting *L. major* at the beginning. However, when THP-1 cells were differentiated with a higher PMA concentration, the initial phagocytic capacities of WT and cGAS-KO cells were comparable and yet by 48 and 72 h, parasite loads in the cGAS knockout cells were significantly lower than the WT cells (**Figures 2C, D**). Furthermore, even though the initial percentage of infected cells and parasite loads in the TBK1-KO cells exceeded that of WT cells (6h, **Figures 2C, D**), absence of TBK1 significantly hindered parasite proliferation at later time points (48 and 72 h). STING knockout cells internalized very little parasites by 6 h and resisted infection throughout all time points.

**Figure 3:**
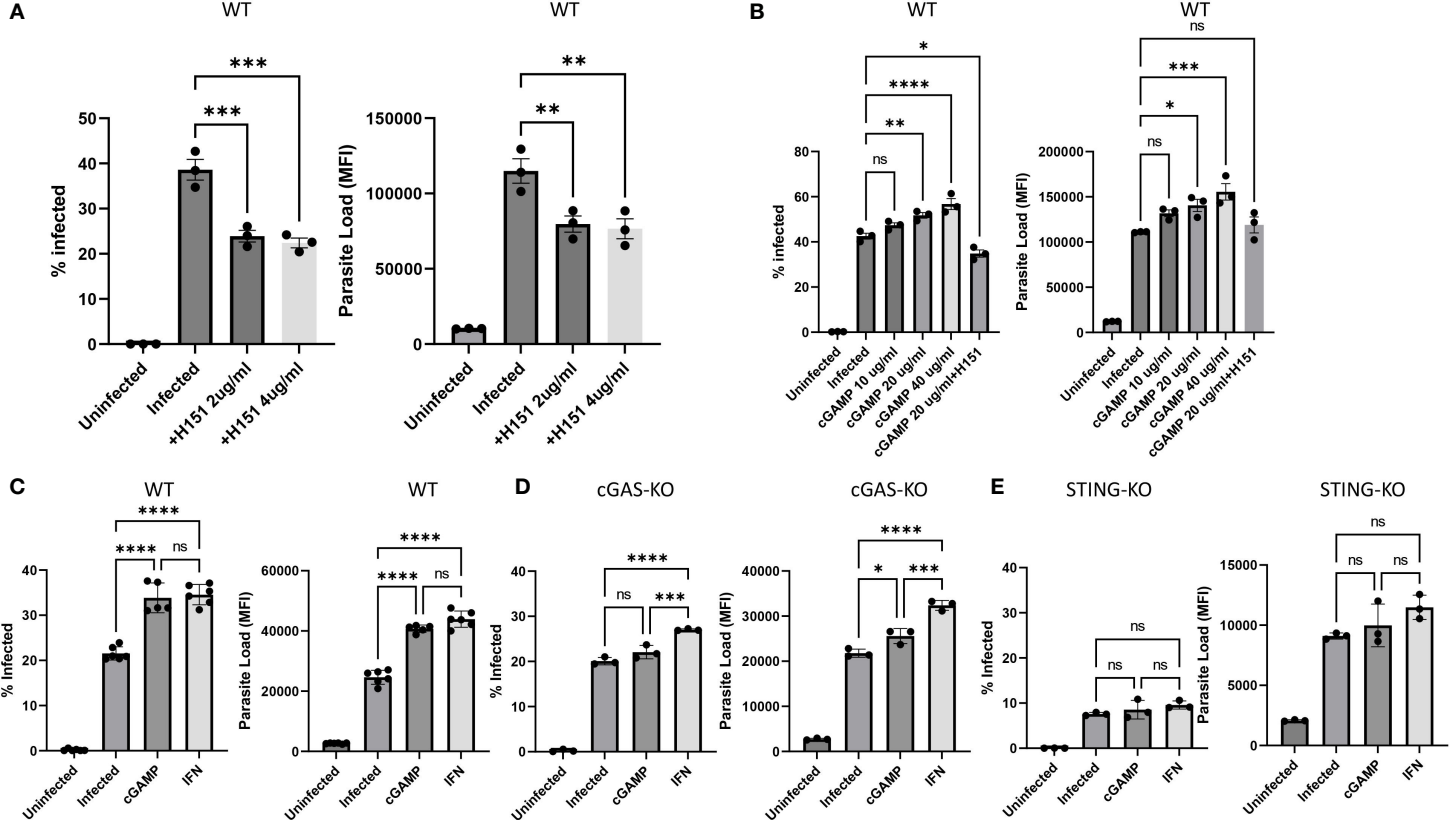
Effect of the STING agonist 2’3’-cGAMP, the STING antagonist H151 and recombinant IFN-β on *in vitro* Leishmania infection. Differentiated WT [**(A)**, **(B)** and **(C)**], cGAS- [**(D)**] or STING- **(E)** knockout THP-1 cells were infected with eGFP expressing L. major parasites at a MOI of 1:10 (macrophage:parasite). Infection percentages and parasite loads were quantified using a flow cytometer 24 h later. H151 [2 or 4 µg/ml; **(A)** and **(B)**], cGAMP [10, 20 or 40 µg/ml; **(B)**, **(C)**, **(D)** and **(E)**] or recIFN-β [50 ng/ml; **(C)**, **(D)** and **(E)**] were added 8, 2 and 8 hours prior to infection, respectively. Data represent average ± SD. All groups were compared statistically by one-way ANOVA followed by Dunnet`s multiple comparison test. ns, not significant, *p≤0.05, **p≤0.01, ***p≤0.001, ****p≤0.0001.

To gain further insight into cGAS/STING related signaling and potentiation of infection, PMA differentiated WT cells were pre-treated either with the STING inhibitor H151 or the STING activator 2’3-cGAMP followed by infection with eGFP expressing transgenic parasites. Compared to untreated WT cells, percentage of infected cells and parasite loads were significantly lower in H151 treated samples (**Figure 3A** and **Supplementary Figure 3A**). In contrast, the STING agonist 2’3-cGAMP dose dependently enhanced both the % infected cells and the parasite loads compared to untreated but infected WT controls (**Figure 3B** and **Supplementary Figure 3B**). Of note, cGAMP (20 µg/ml) failed to potentiate infection in the presence of the inhibitor H151 (**Figure 3B** and **Supplementary Figure 3B**). Comparison of infected versus infected/cGAMP-treated WT, cGAS- and STING-knockout cells demonstrated that the STING agonist enhanced infected cell percent in WT cells and parasite loads in WT and cGAS- but not in STING-knockout cells (**Figures 3C–E** and **Supplementary Figures 3C–E**). Furthermore, exogenous recombinant IFNβ supplementation also enhanced infection rate and parasite loads (**Figures 3C–E** and **Supplementary Figures 3C–E**). Collectively, these results suggest that leishmania parasites might hijack the cGAS-STING-TBK1 signaling pathway to their own advantage.

### Gene expression analysis in infected WT versus cGAS and TBK1 knockout cells

**Figure 4:**
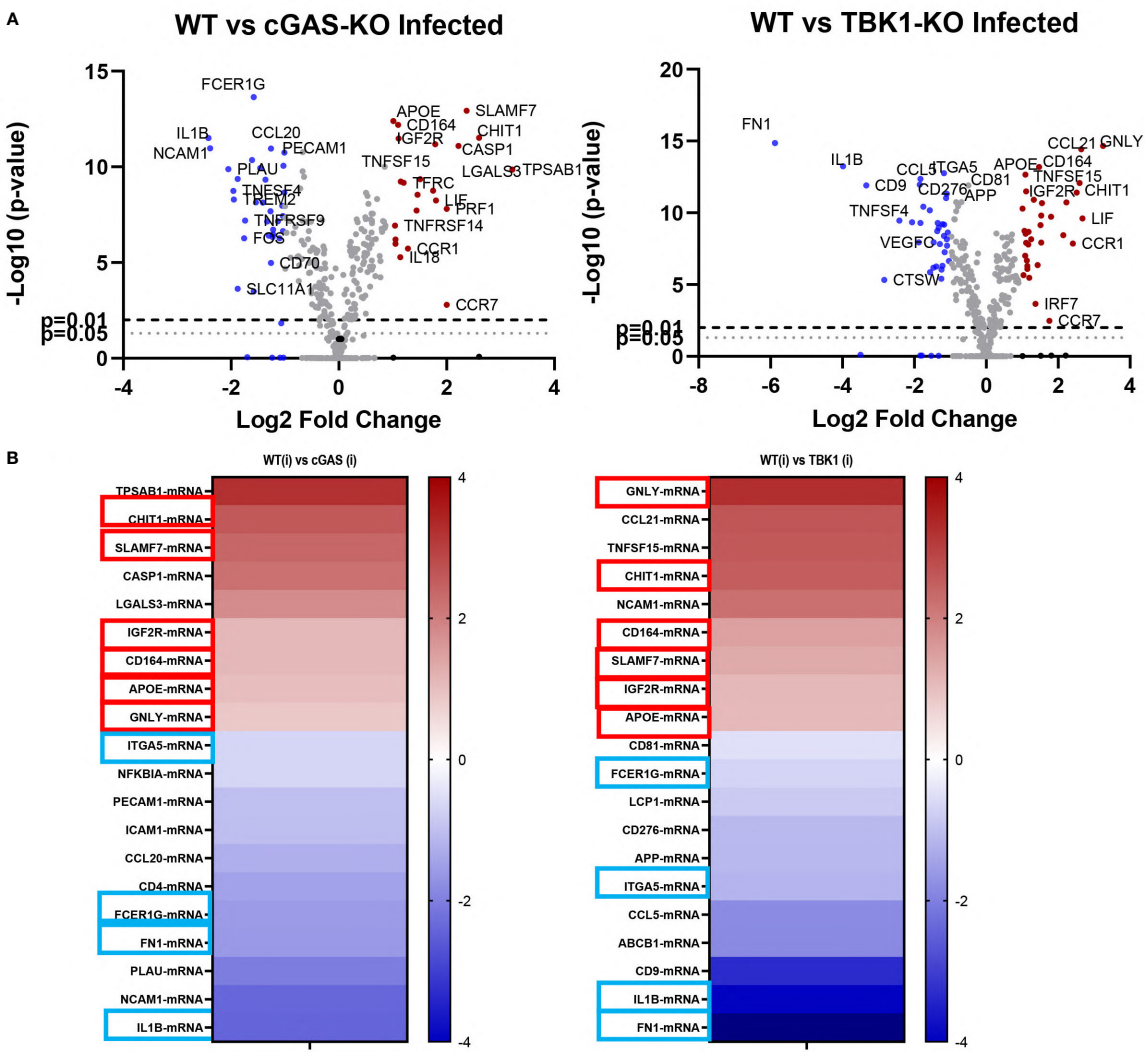
Differentially expressed genes in infected WT versus cGAS and TBK1 knockout cells. **(A)** Volcano plots of differentially expressed genes between *L. major* infected WT and knockout cell lines (n=3). Upregulated (fold change ≥ 2) and downregulated (fold change ≤ 2) genes are shown in red and blue, respectively. Dashed lines represent p=0.01 and dotted lines show p=0.05. The log2FC is plotted on the x-axis, and the negative log10FDR (adjusted p-value) is plotted on the y-axis. **(B)** Heatmaps showing the top 10 most up- or down-regulated genes in the infected knockout cells with respect to infected WT controls. Six of the shared upregulated genes and 4 of the shared downregulated genes are enclosed in boxes (red boxes, upregulated; blue boxes, downregulated). Statistical analysis for differentially expressed genes were performed using nSolver Analysis software (v4, NanoString Technologies, Inc., U.S.A.) and nCounter^®^ Advanced Analysis Software (v2, NanoString Technologies, Inc., U.S.A.).

To gain further insight into the intrinsic resistance of knockout cell lines to leishmania infection, we analyzed the gene expression profiles of WT, cGAS and TBK1 knockout cells using the NanoString nCounter PanCancer Immune Profiling CS Kit. For this, RNAs were extracted from cells 48 h-post-infection. Volcano plots comparing the fold changes in expression (Log2) with the corresponding adjusted p-values (−log10) in infected knockout cells in comparison to infected WT cells are shown in **Figure 4A**.

Of the 770 genes found in the panel, 24 and 37 genes were differentially up- and downregulated in infected cGAS knockout cells when compared to infected WT cells, respectively (**Figure 4A**, left plot). In the TBK1 knockout cells, 37 genes were upregulated and 39 downregulated when compared to infected WT cells (**Figure 4A**, right plot). Out of the top ten most up- or down-regulated genes in both knockout cells, six of the upregulated and four of the downregulated genes were identical (**Figure 4B**). Granulysin (GNLY), Chitotriosidase-1 (CHIT1), Sialomucin core protein 24 (CD164), SLAM Family Member 7 (SLAMF7), insulin-like growth factor receptor 2 (IGF2R) and apolipoprotein E (APOE) genes were upregulated in infected cGAS and TBK1 knockout cell lines in comparison to infected WT controls. The four shared down-regulated genes were Fc Epsilon Receptor Ig (FCER1G), Integrin Subunit Alpha 5 (ITGA5), Interleukin 1 beta (IL1B) and Fibronectin 1 (FN1). Collectively, these commonly up- and down-regulated genes might play a role in modulating the response of cells during infection with leishmania parasites.

### The effect of TBK1 inhibitor on Leishmania infection

Since TBK1 knockout cells were highly resistant to infection, we next wanted to evaluate the effect of a TBK1 inhibitor on leishmania infection in WT cells. For this, WT THP-1 cells were pre-treated with increasing concentrations of amlexanox prior to a 24-hour *L. major* infection assay (**Supplementary Figure 4**). Amlexanox treatment potently inhibited both the percentage and the parasite load of infected cells in a dose dependent manner. Similarly, another TBK1 inhibitor, BX795, also significantly reduced parasite loads in infected cells (**Supplementary Figure 5**), although this decrease was less pronounced when compared to amlexanox-induced infection suppression.

**Figure 5:**
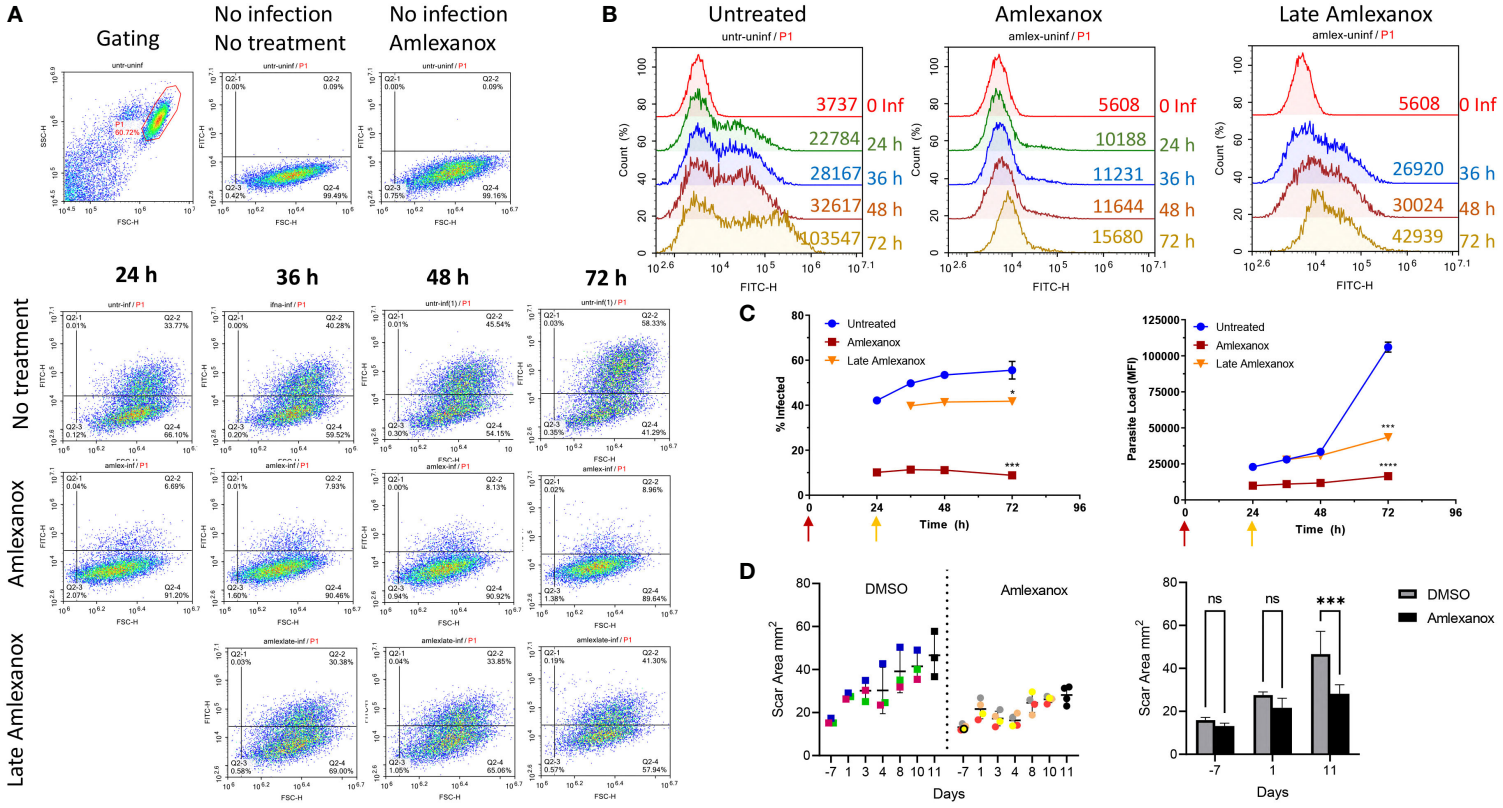
Amlexanox-induced inhibition of L. major infection in WT THP1 cells in a 3-day infection model. Differentiated THP-1 cells were infected with eGFP expressing *L. major* parasites at a MOI of 1:10 (macrophage:parasite). Infection percentages and parasite loads were quantified using a flow cytometer at the indicated time points. Amlexanox was added either 1 hour prior to infection or 24 h post infection (late amlexanox). **(A)** and **(B)** show representative flow cytometry density and histogram plots, corresponding to infected cell percent and parasite load, respectively. **(C)** Data presented in graphs are representative of two independent experiments with similar results Amlexanox was added at different time points in treated groups (purple, 1 h pre-infection; red, 24 h post-infection). 72 h data were statistically compared to untreated group using one-way ANOVA followed by Bonferroni’s multiple comparison test. **(D)** Footpad swelling in *L. major* infected mice treated or not with amlexanox starting on day 41 post-infection for or a duration of 12 days. Results were based on digital caliper measurement of footpad depth and width of DMSO injected controls (n=3) and amlexanox injected (n=4) treatment group. Data were statistically compared using multiple unpaired t tests. ns, not significant, *p≤0.05, ***p≤0.001, ****p≤0.0001.

The effect of amlexanox on Leishmania infection was further analyzed in a 3-day *in vitro* infection model and an *in vivo* therapeutic treatment model (**Figure 5**). As expected, amlexanox addition to THP-1 cells 1 hour prior to infection, potently inhibited both the infection rate and proliferation of *L. major* parasites (**Figures 5A-C**, purple line). To evaluate whether amlexanox could exert an inhibitory effect on already infected cells, the drug was added to cells 24 h-post infection. The results showed that there was still a significant inhibitory effect of late amlexanox addition on infected cell percent and parasite load by 72 h (**Figures 5A-C**, red line).

Since *in vitro* infection experiments revealed a potent anti-parasitic effect of amlexanox treatment, we wanted to assess the drug’s anti-leishmanial activity *in vivo*. For this, seven mice were infected with *L. major* and on day 41 post-footpad challenge, the drug (n=4) or DMSO control (n=3) was injected to mice on 12 consecutive days. Throughout the therapy period footpad swelling was monitored by a digital caliper (**Figure 5D**). Days presented in the Figures correspond to the initiation and continuation of amlexanox therapy. Results demonstrated that amlexanox treatment delayed footpad swelling ˜2-fold (30.31% change between days 1 and 11), when compared to the DMSO control group (69.14% change between days 1 and 11) (**Figure 5D**). These results collectively suggest that amlexanox could be a new candidate drug for treatment of cutaneous leishmaniasis caused by *L. major*.

## Discussion

Multiple pattern recognition receptors have been demonstrated to be involved in the modulation of host response during the course of leishmania infections (41–45). Yet, the role of the cGAS-STING DNA sensing pathway in regulation of leishmania infections remains largely unexplored. In this study, we revealed a critical role of the cGAS-STING-TBK1 signaling pathway in modulating parasite loads in infected macrophages. The cGAS-STING-TBK1 pathway was previously shown to be exploited by non-viral pathogens to favor their persistence. For example, *Listeria monocytogenes*, *Staphylococcus aureus* and *Neisseria gonorrhea* exploit the cGAS-STING–TBK1 pathway to impair antibacterial defenses, promoting bacterial persistence (46–48). With respect to infections caused by protozoan parasites, stimulation of the cGAS-STING-TBK1 pathway can yield detrimental or protective outcomes (26). For example, one study demonstrated that hijacking of the cGAS-STING-TBK1-IRF3 pathway by *Toxoplasma gondii*, promoted its replication both *in vitro* and *in vivo* (30). In contrast, another study found a protective effect of cGAS, STING and type-I interferons during *Toxoplasma gondii* infection (49).

Evidence suggests that cGAS preferentially binds to U-turns or bent DNA helices which favor dimerization and nucleation of protein-DNA ladder formation (39). In this context, bent DNA was first discovered due to electrophoretic anomalies exhibited by the AT-rich kDNA of kinetoplastid parasites (40, 50). Therefore, it is highly likely that the affinity of cGAS towards optimally pre-structured kDNA would be much higher in magnitude when compared to linear genomic DNA. Consistent with this view, our results demonstrated that kDNA delivery to the cytosol magnified parasite burden of infected macrophages. Whether these unique DNA networks from different kinetoplastida also share a role of modulating host defenses remains to be explored.

Our data on gene expression analysis in infected cGAS and TBK1 knockout cell lines identified a number of shared up- and down-regulated genes which collectively might contribute to establishment of infection resistance. Among the shared up-regulated genes, SLAMF7 stands out as a receptor linked to superactivated macrophage phenotype (51). Similarly, granulysin and chitotriosidase expression in macrophages were associated with microbial defense (52, 53). Among the downregulated genes, ITGA5 (integrin subunit alpha 5) stands out since the protein encoded by this gene is a constituent of fibronectin receptor (integrin α5β1) which has a prominent role in promastigote internalization (54).

Having established that cGAS-STING-TBK1 axis plays an important role in regulation of parasite burden in infected macrophages, we next asked whether a TBK1 inhibitor could be of benefit in inhibiting parasitemia *in vitro* and *in vivo*. Indeed amlexanox, a specific inhibitor of IKKϵ and TBK1 proved to be highly effective in controlling parasite loads both *in vitro* and *in vivo*. This result merits further investigation on the potential of amlexanox as a surrogate treatment strategy for cutaneous leishmaniasis. In summary, our results imply that kDNA of leishmania parasites potentially engage cGAS and modify the response of host cells in a STING- and TBK1-dependent manner to promote a pro-parasitic state. Future studies investigating the role of cGAS-STING-TBK1 pathway using knockout mouse models of leishmania infection and research on inhibitors of this pathway should unravel specific mechanisms involved in leishmania-mediated immune evasion.

## Data availability statement

The data presented in the study are deposited in the Gene Expression Omnibus (GEO) repository, accession number GSE211590. Data is available through the following link: https://www.ncbi.nlm.nih.gov/geo/query/acc.cgi?acc=GSE211590.

## Ethics statement

All protocols carried out in this study were approved by animal ethics committee of Bilkent University (Protocol No: BILAEC-2019/44).

## Author contributions

IY, ED, IA, IG and MG designed the study, analyzed the data and wrote the manuscript. EI, MY and GA assisted experiments. NG and AO provided leishmania parasites and shared culture conditions, YO and ST provided leishmania parasites and shared kinetoplast DNA isolation protocol. All authors contributed to the article and approved the submitted version.

## Funding

This work is supported by TUBITAK grants 115S073 and 219S678 to MG.

## Acknowledgments

We thank Dr. Deniz Kahraman of Middle East Technical University, Informatics Institute for technical help with the nCounter Nanostring assay.

## Conflict of interest

The authors declare that the research was conducted in the absence of any commercial or financial relationships that could be construed as a potential conflict of interest.

## Publisher’s note

All claims expressed in this article are solely those of the authors and do not necessarily represent those of their affiliated organizations, or those of the publisher, the editors and the reviewers. Any product that may be evaluated in this article, or claim that may be made by its manufacturer, is not guaranteed or endorsed by the publisher.
