# Supplementary material for: Leishmania kinetoplast DNA contributes to parasite burden in infected macrophages: Critical role of the cGAS-STING-TBK1 signaling pathway in macrophage parasitemia
Source: Front Immunol. 2022 Nov 2;13:1007070. doi: 10.3389/fimmu.2022.1007070 (PMC9667060; doi:10.3389/fimmu.2022.1007070)
Supplement: Supplementary file 1 [file Image_1.pdf]

Supplementary Figures

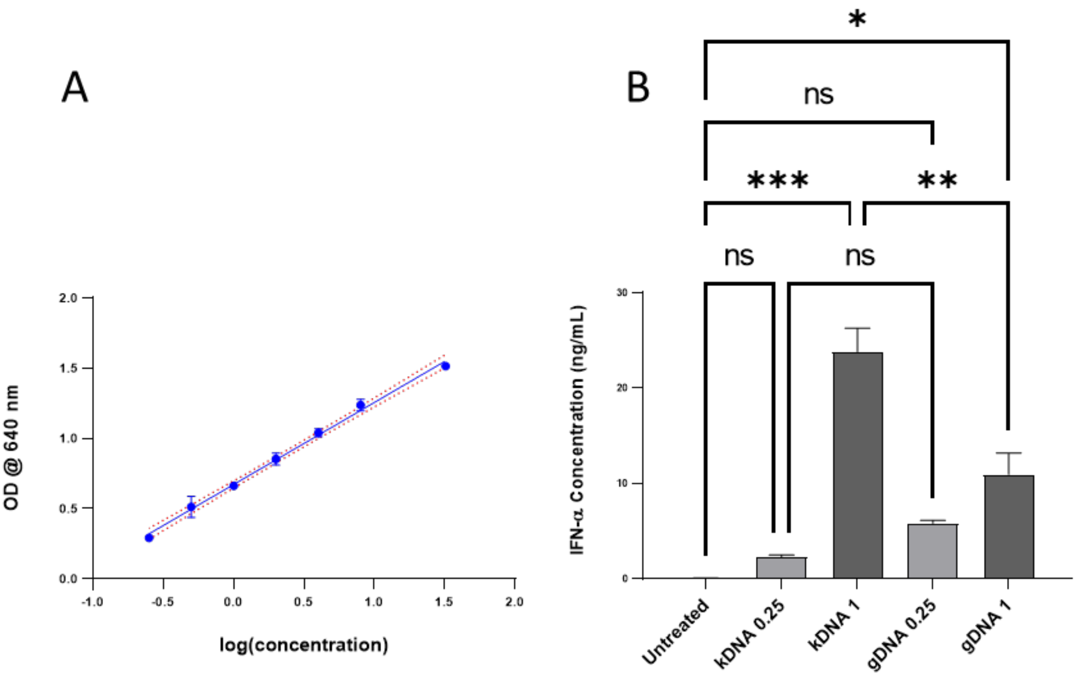

Supplementary Figure 1. Response of infected THP-1 cells to cytosolic delivery of DNA. Type-I IFN production (as a specific readout of the actual response) in PMA-differentiated THP-1 blue cells upon kDNA or Leishmania genomic DNA (gDNA) stimulations were measured by the colorimetric Quanti-Blue assay based on alkaline phosphatase activity in supernatants. To construct a four parametric logistic standard curve for IFN concentration estimation, OD values obtained from supernatants of uninfected PMA-differentiated THP-1 cells stimulated with different concentrations (0.25-32 ng/ml) of recombinant IFN-α were used (**A**). Using this standard curve, OD values obtained from supernatants of infected cells stimulated with kDNA or gDNA were used to calculate concentration of IFN produced by stimulated cells (**B**).

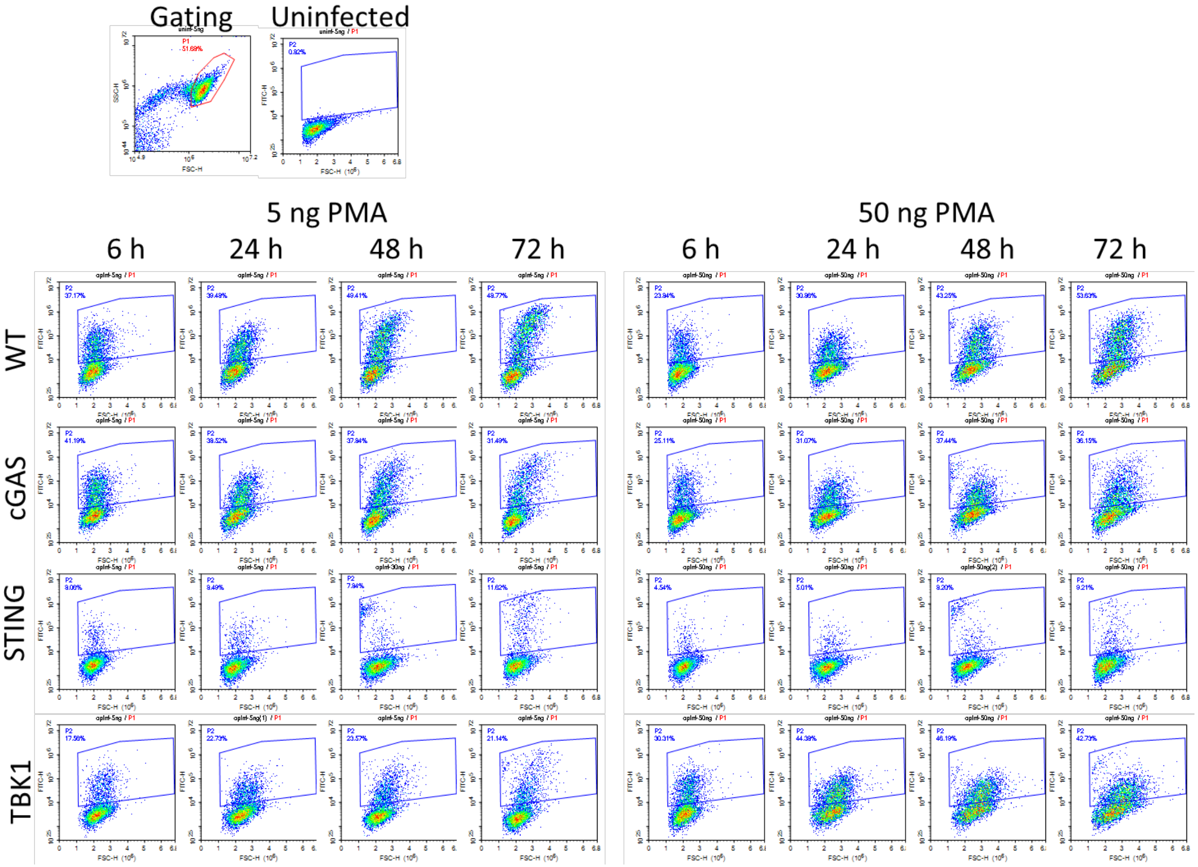

Supplementary Figure 2. Kinetics of Leishmania infection in WT versus knockout THP-1 cells. Low dose (5 ng) or high dose (50 ng) PMA differentiated THP-1 cells were infected with eGFP expressing *L. major* parasites at a MOI of 1:10 (macrophage:parasite). At indicated time points, infection percentages were quantified by flow cytometry.

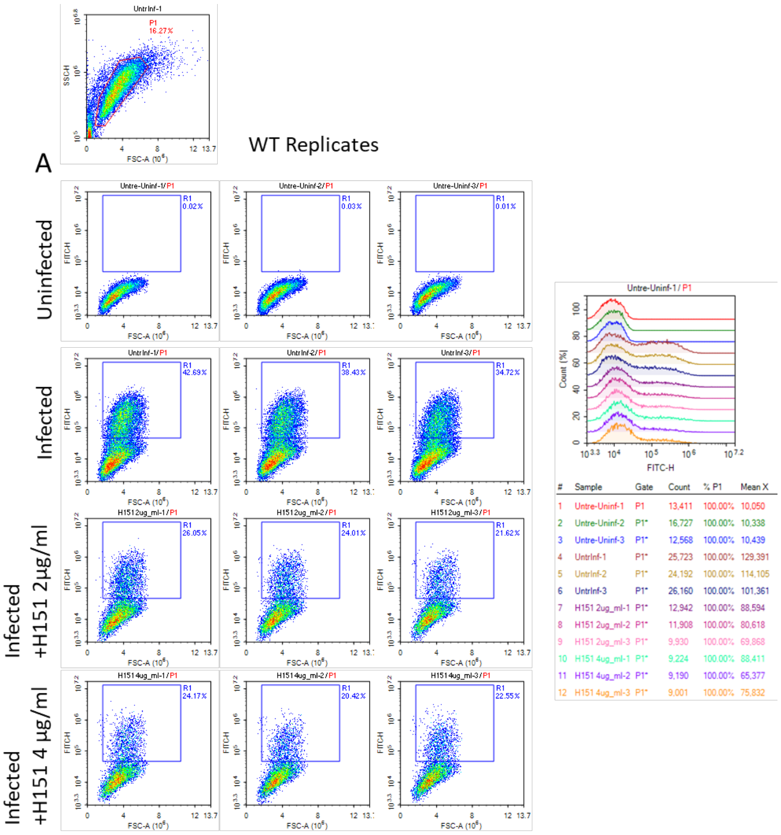

#	Sample	Gate	Count	% P1	Mean X
1	Untre-Uninf-1	P1	13,411	100.00%	10,050
2	Untre-Uninf-2	P1*	16,727	100.00%	10,338
3	Untre-Uninf-3	P1*	12,568	100.00%	10,439
4	UntrInf-1	P1*	25,723	100.00%	129,391
5	UntrInf-2	P1*	24,192	100.00%	114,105
6	UntrInf-3	P1*	26,160	100.00%	101,361
7	H151 2ug_ml-1	P1*	12,942	100.00%	88,594
8	H151 2ug_ml-2	P1*	11,908	100.00%	80,618
9	H151 2ug_ml-3	P1*	9,930	100.00%	69,868
10	H151 4ug_ml-1	P1*	9,224	100.00%	88,411
11	H151 4ug_ml-2	P1*	9,190	100.00%	65,377
12	H151 4ug_ml-3	P1*	9,001	100.00%	75,832

Supplementary Figure 3A. Effect of the STING antagonist H151 on in vitro Leishmania infection. Differentiated Wild Type (WT) THP-1 cells were infected with eGFP expressing L. major parasites at a MOI of 1:10 (macrophage:parasite). H151 treatments were 2 h prior to initiation of infections. Infection percentages (density plots) and parasite loads (Histogram plot) were quantified from triplicate samples using a flow cytometer 24 h later.

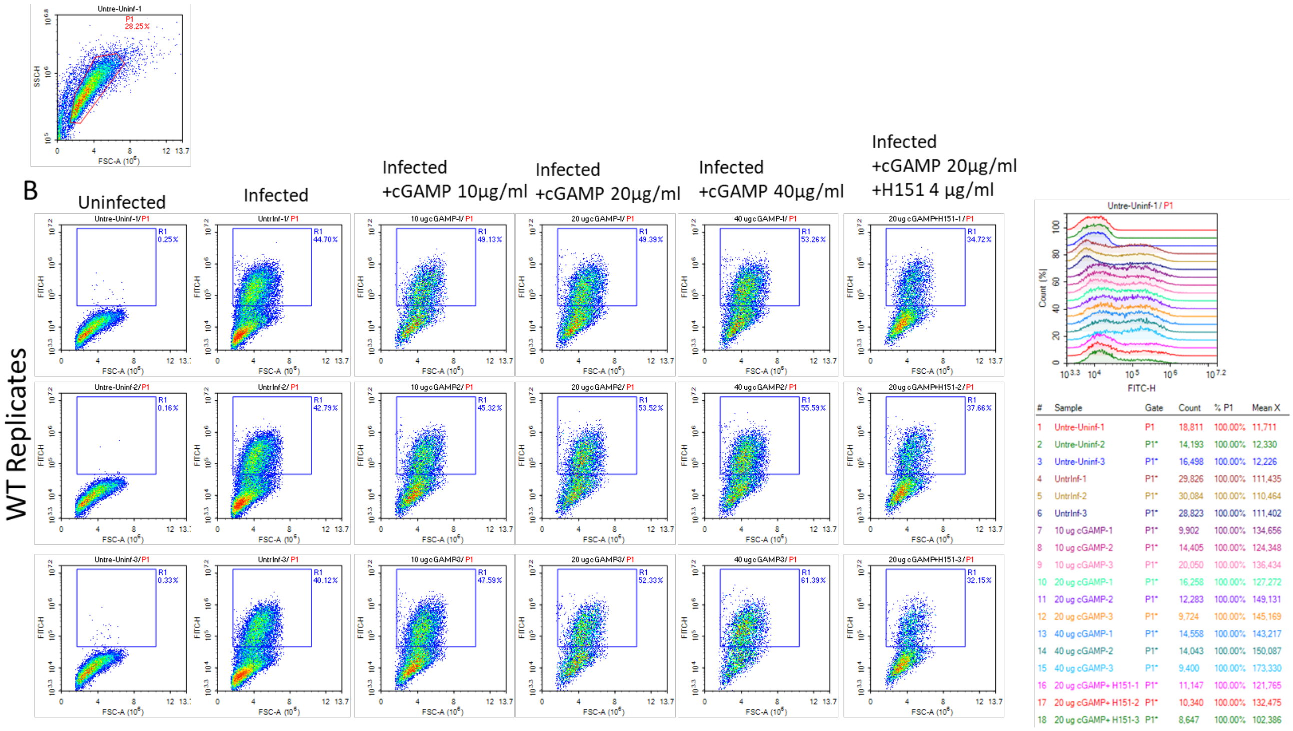

Supplementary Figure 3B. Effect of the STING agonist 2’3’-cGAMP on in vitro Leishmania infection. Differentiated (50 ng/ml PMA) Wild Type (WT) THP-1 cells were infected with eGFP expressing L. major parasites at a MOI of 1:10 (macrophage:parasite). 2’3’-cGAMP treatments were 8 h prior to initiation of infections. Infection percentages (density plots) and parasite loads (Histogram plot) were quantified from triplicate samples using a flow cytometer 24 h later.

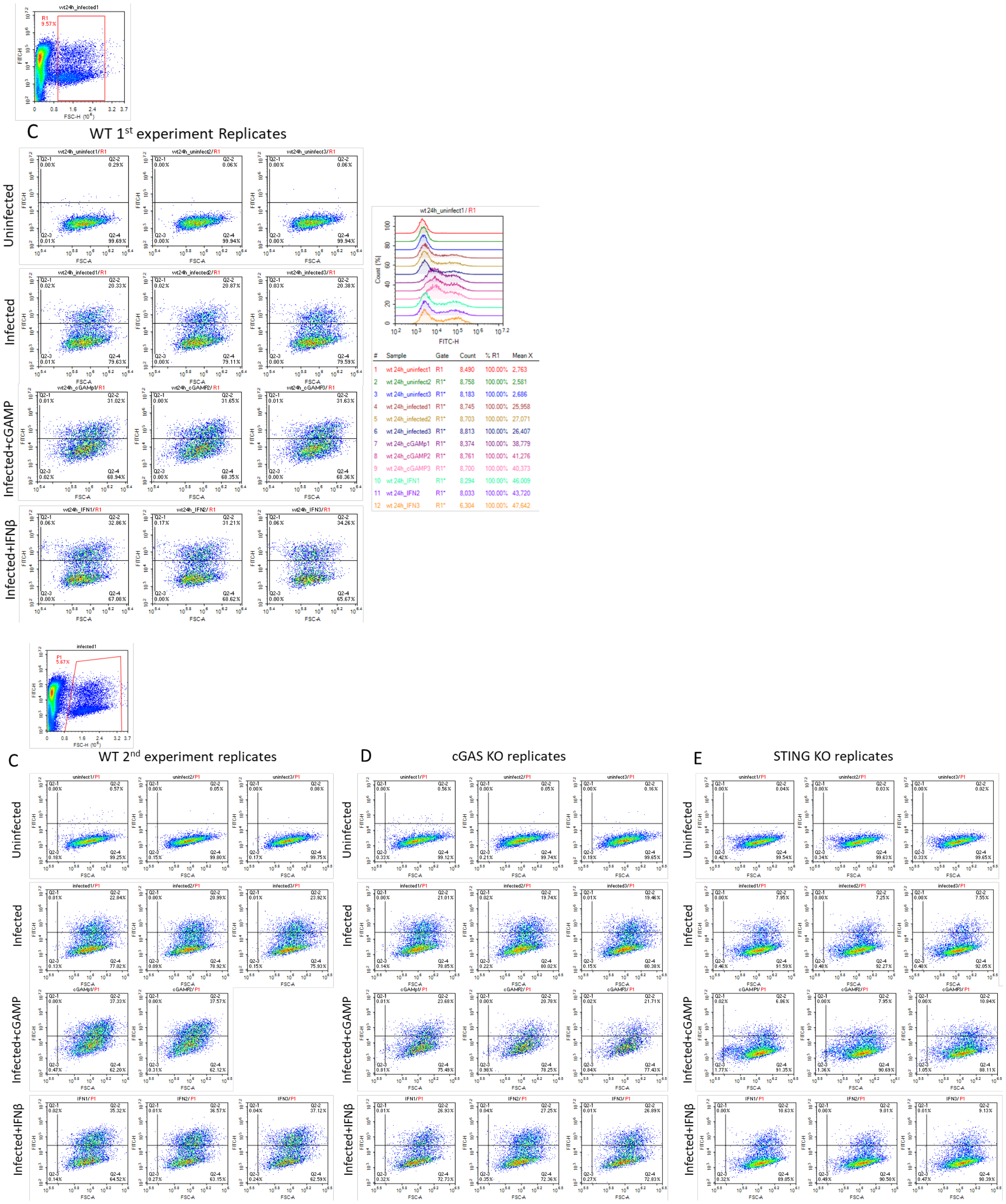

Supplementary Figure 3C, D and E. Effect of the STING agonist 2’3’-cGAMP and recombinant IFNβ on in vitro Leishmania infection. Differentiated (50 ng/ml PMA) Wild Type (WT) (**C**), cGAS knockout (**D**) and STING knockout (**E**) THP-1 cells were infected with eGFP expressing L. major parasites at a MOI of 1:10 (macrophage:parasite). 2’3’-cGAMP (20 µg/ml) and recombinant IFNβ (50 ng/ml) treatments were 8h and 2 h prior to initiation of infections, respectively. Infection percentages (density plots) and parasite loads (Histogram plot) were quantified from 5-6 samples (WT) or triplicate samples (cGAS- and STING- knockout) using a flow cytometer 24 h later.

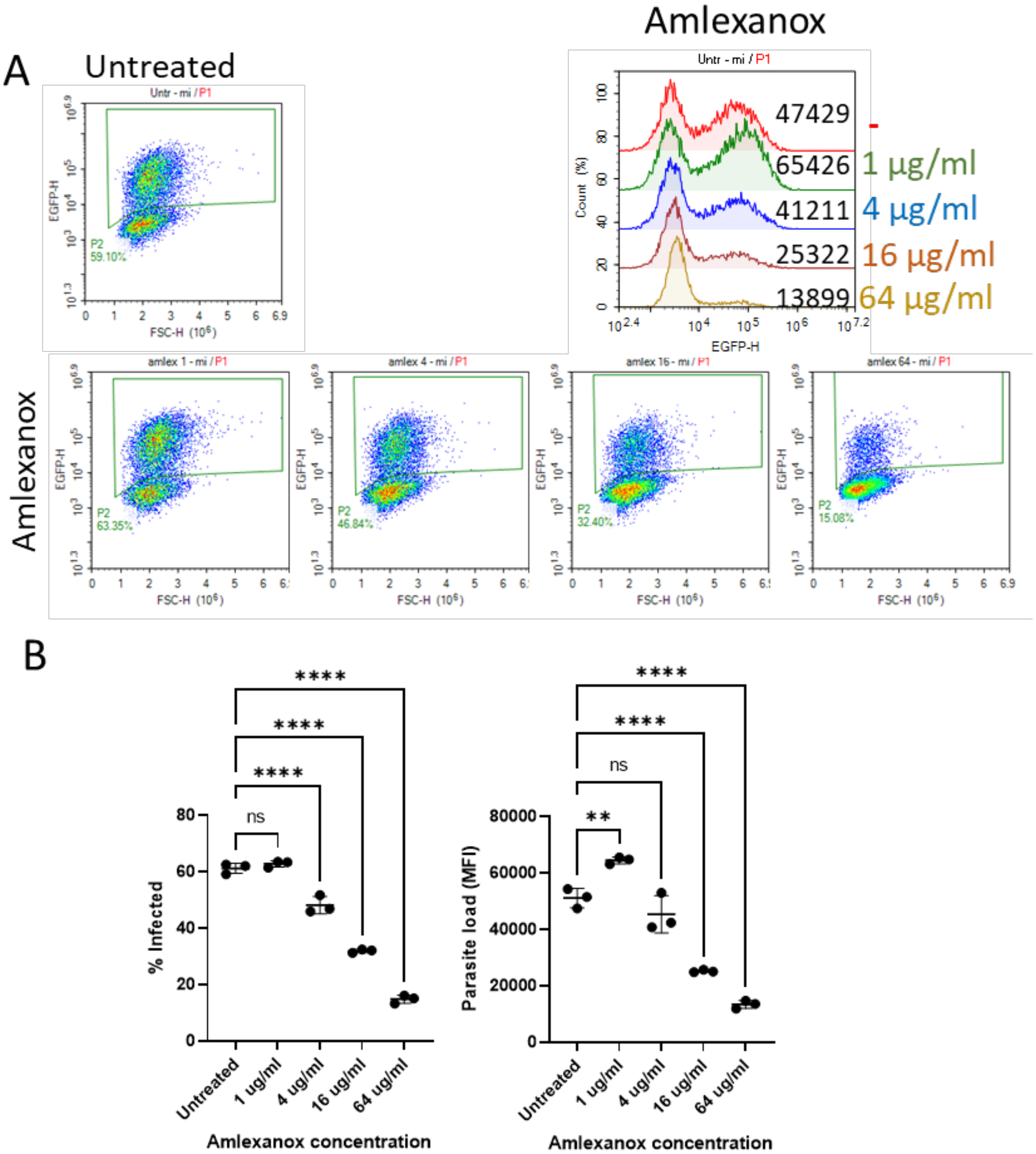

Supplementary Figure 4. Effect of increasing doses of Amlexanox on *L. major* infection. Differentiated (50 ng/ml) WT THP1 cells were pre-treated with increasing doses of Amlexanox for 2 hour. eGFP expressing *L. major* parasites were co-incubated with THP-1 cells for 24 hours. Infection percentages and parasite loads were quantified using a flow cytometer.

Treatment groups were statistically compared to untreated group using one-way ANOVA followed by Dunnet’s multiple comparison test.

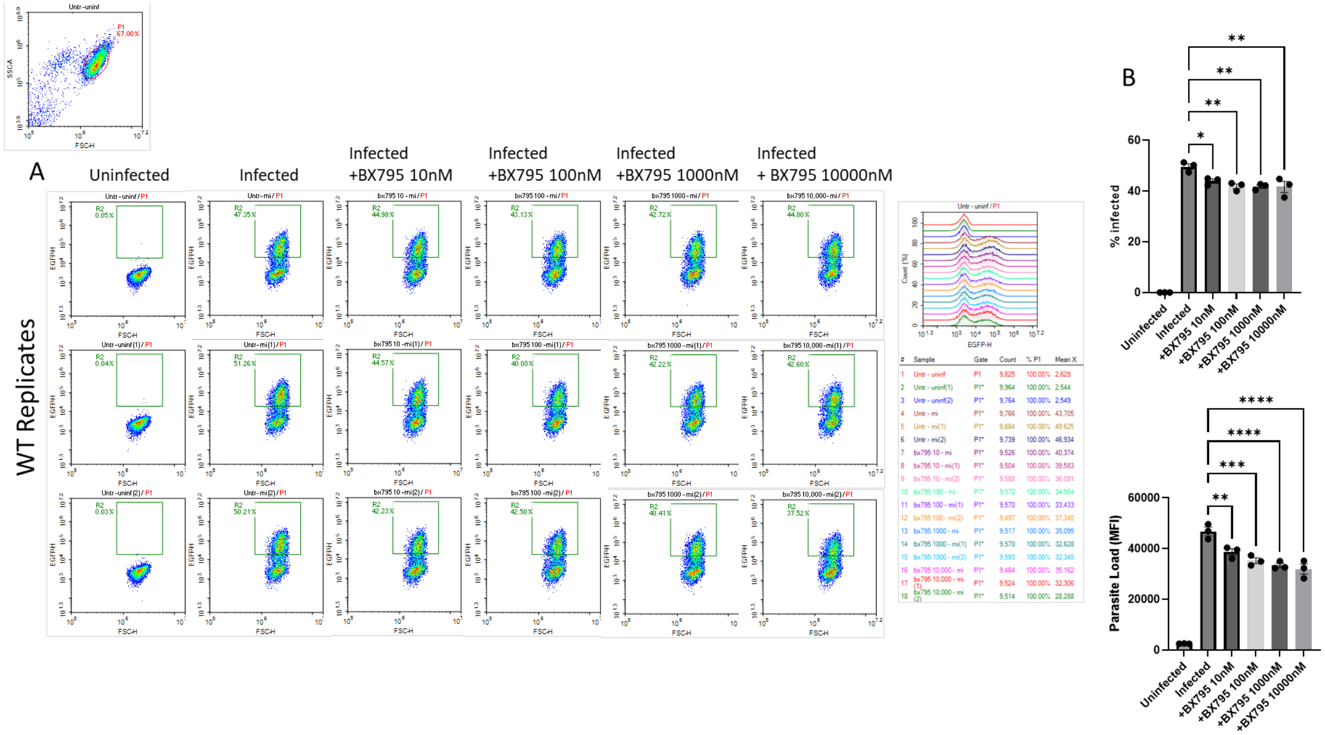

Supplementary Figure 5. Effect of increasing doses of BX795 on *L. major* infection. Differentiated (50 ng/ml) WT THP1 cells were pre-treated with increasing doses of BX795 for 2 hours. eGFP expressing *L. major* parasites were co-incubated with THP-1 cells for 24 hours. Infection percentages and parasite loads were quantified using a flow cytometer. Treatment groups were statistically compared to untreated group using one-way ANOVA followed by Dunnet’s multiple comparison test.
